# Survival analysis of extramammary Paget’s disease (EMPD) in a tertiary hospital in Taiwan

**DOI:** 10.1186/s12957-021-02228-z

**Published:** 2021-04-12

**Authors:** Yu-Wei Chang, Hsu Ma, Wen-Chieh Liao

**Affiliations:** 1 Division of Plastic and Reconstructive Surgery, Department of Surgery, Taipei Veterans General Hospital, 19F, No.201 Shih-Pai RD Sec 2, Taipei, Taiwan; 2 School of Medicine, National Yang-Ming University, Taipei, Taiwan; 3 Institute of Environmental and Occupational Health Sciences, School of Medicine, National Yang Ming Chiao Tung University, Taipei, Taiwan

**Keywords:** Extramammary Paget disease (EMPD), Survival analysis, Metastasis wide excision

## Abstract

**Background:**

This study aimed to investigate the survival analysis of extramammary Paget’s disease (EMPD) in a Taiwanese population and to provide data for comparison with other studies in various locations and racial populations.

**Methods:**

We retrospectively analyzed the medical records of 63 patients with EMPD who were surgically treated from 2002 to 2019 at a single institution. The primary endpoint was the 5-year overall survival rate of EMPD, and the secondary endpoint was recurrence-free 5-year survival. Independent variables included patients’ demographic data, concurrent malignancy (i.e., non-EMPD-related cancers), tumor size, distant metastasis, and surgery and/or radiation.

**Results:**

Of all the 63 patients, 8 cases were excluded. A total of 43 patients (78.18%) were male, and 12 were female, with a mean age of 72.67 years (range 44–89 years). The most common affected anatomic site was the penoscrotal region (22 patients, 40.00%), followed by the perianal and perineal regions (17 patients, 30.91%). Among the 55 patients, 41 patients (74.55%) were diagnosed with at least one underlying disease, whereas the most common underlying disease was cardiovascular disease (30 patients, 54.55%). The overall survival rate was 80.00% at 36 months and 65.45% at the end of follow-up. EMPD with deep dermal invasion was a significant poor prognostic factor of overall survival in cause-specific hazard model (sub-hazard ratio (HR) 5.167, *p* = 0.0015, 95% confidence interval (CI) 1.876–14.230). Patients with regional metastasis or distant metastasis had poorer prognosis of 5-year survival (sub-HR 4.513, *p* = 0.0028, CI 1.683–12.103). The limitations of this study include its retrospective nature and sample size.

**Conclusions:**

In our series, EMPD with metastasis and deep dermal invasion was the significant harmful factors in both overall 5-year survival and 5-year recurrence-free survival. The surgical excision is not associated with a low risk of local recurrence or overall survival, and long-term follow-up is still needed.

## Background

Extramammary Paget’s disease (EMPD) is a rare intraepithelial neoplasm that most commonly affects individuals in their 60s to 80s [1, 2]. Given its slow growth and non-specific symptoms, EMPD is easily neglected and results in delayed diagnosis [2–4]. The disease affects sites rich in apocrine glands, including the vulva, scrotum, penis, and perineal and perianal regions and less frequently in the axilla, face, or trunk. High prevalence in Caucasians and predominance in female were reported in Western literature, whereas less frequent occurrence was reported for Asian populations [1–4].

Previous literatures have identified potential factors related to poor prognosis of EMPD; these factors include the dermis invasion, distant metastasis, concurrent malignancy, male gender, and tumor in the perianal anatomic region [2–6]. Karam et al. conducted a survival analysis of white people-predominant population with 2001 EMPD patients in 1973–2007 and concluded the high mortality in invasive EMPD patients with old age, advanced stage, and treatment modality [4].

Different characteristics and manifestations of EMPD in Asian population, including male predominance and low incidence of concurrent internal malignancy, have been identified [7, 8]. Nevertheless, given the relative rarity of EMPD in Asian population, limited literature reported findings on Taiwanese population [8–11], whereas a similar comprehensive survival analysis in Taiwan is still lacking.

In this study, we presented our 18-year experience of EMPD cases in a single center in Taiwan. We aimed to analyze the demographic characteristic of the disease and identify potential prognostic factors of overall survival and recurrence-free survival in Taiwanese population.

## Methods

### Patient selection and inclusion criteria

This retrospective cohort study was conducted by the plastic surgery department of Taipei Veterans General Hospital, Taiwan. The study was approved by the institutional review board of our hospital. Through the electronic patient record system, in January 2002 to January 2019, patients who received biopsy with final diagnosis of EMPD on pathological reports were included. The 5-year survival status was confirmed through electronic patient records. If the survival status cannot be confirmed, phone interview was performed.

### Data extraction and selection

Patient demographic characteristics, including age of diagnosis, gender, concurrent malignancy, anatomic site of lesion, maximal diameter of lesion, and metastasis status, were extracted and recorded. Dermal invasion of the lesion was divided into upper dermis invasion and deep dermal invasion, and the metastasis status was further classified as regional or distant metastasis. The type of treatment was classified into four groups, including surgical excision alone, surgical excision with adjuvant therapy, nonsurgical treatment alone (radiotherapy, chemotherapy, or phototherapy), and without any treatment. In addition to wide local excision, simple or radical vulvectomy in vulva EMPD was included in the excision. Surgical outcomes, including status of excision margin, recurrence, and recurrence-free interval, were also recorded.

### Primary and secondary endpoints

The primary endpoint was the 5-year overall survival rate of EMPD, which was defined as the interval between the date of diagnosis on pathology to the date of death of any cause. The poor prognostic factors of 5-year overall survival were identified. The secondary endpoint was recurrence-free 5-year survival, defined as the interval between the date of diagnosis on pathology to the date of recurrence or death of any cause. The related risk factors of recurrence were also analyzed.

### Statistical analysis

All the data were analyzed by the SAS® 9.4 software. Discrete variables were presented in percentages, and the continuous variables were presented as mean and standard deviation. Competing risk analysis with cause-specific hazard model was applied to evaluate the variables individually to identify the potential factors of poor prognosis in both 5-year overall survival and recurrence. The proportional hazard assumption of the cause-specific hazard model would be tested. Significance was set at *p* ≤0.05 for each test.

## Results

**Fig. 1:**
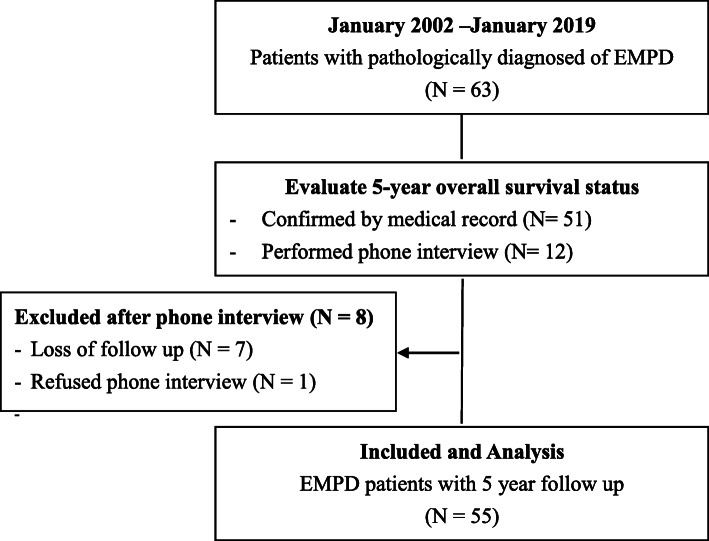
Data extraction and exclusion and inclusion criteria of our series

Between January 2002 and January 2019, 63 patients were diagnosed with EMPD in our hospital. To evaluate the 5-year overall survival status, in addition to the electronic medical record, phone interviews were performed to twelve patients, whereas seven patients were lost to follow-up and one refused the phone interview (Fig. 1). Table 1 lists the demographic characteristics of 55 eligible patients. The mean age diagnosis was 72.67 years (range 44–89 years), with 30 (54.55%) patients diagnosed at 75 years old or older. The majority of the diagnosed patients were male (43 patients, 78.18%), and the most common affected anatomic site was the penoscrotal region (22 patients, 40.00%). The second most affected region was the perianal and perineal region (17 patients, 30.91%). More than half of the patients were diagnosed with a lesion larger than 2 cm (36 patients, 65.45%). Among the 55 patients, 41 (74.55%) were diagnosed with at least one underlying disease, whereas the most common underlying disease was cardiovascular disease (30 patients, 54.55%), followed by metabolic or endocrine diseases (15 patients, 27.27%).

**Table 1:** Demographics and clinical data of 55 study patients with EMPD

Variable	Patients
Patient characteristics	
Gender (male)	
Male	43 (78.18%)
Female	12 (21.82%)
Age (year) (mean= 72.67, range 44–89)	
Age < 65 years old	14 (25.45%)
Age= 65–74 years old	11 (20.00%)
Age = 75 years old or more	30 (54.55%)
Anatomic site of lesion	
Scrotum or penis	22 (40.00%)
Vulva or labia	7 (12.73%)
Perianal or perineal region	17 (30.91%)
Trunk or others	9 (16.36% )
Types of treatment	
Surgical excision only	39 (70.91%)
Surgical excision with adjuvant therapy	8 (14.55%)
Radiotherapy or chemotherapy only	4 (7.27%)
Refused any treatment	4 (7.27%)
Recurrence (*N*=51)	8 (15.69%)
Concurrent malignancy	(*N*=21, 38.18%)
Adnexal carcinoma	3 (5.46%)
Internal malignancy	18 (32.73%)
Underlying diseases	
Without any underlying diseases	14 (25.45%)
Cardiovascular diseases	30 (54.55%)
Respiratory diseases	5 (9.09%)
Metabolic or endocrine diseases	15 (27.27%)
Nephrology disease	3 (5.45%)
Gastrointestinal disease	8 (14.55%)
Pathological parameter	
Size of lesion (length of maximal diameter)	
2 cm or less than 2 cm	19 (34.55%)
More than 2 cm	36 (65.45%)
Depth of invasion	
Intraepithelial	38 (69.09%)
Micro-invasion of upper dermis	10 (18.18%)
Deep invasion	7 (12.73%)
**Immunohistochemical staining (*N*=36)**	
**Positive of CK7**	**33 (91.67%)**
**Positive of CK20**	**8 (22.22%)**
**Positive of GCDFP-15**	**5 (13.89%)**
**Positive of Cdx2**	**8 (22.22%)**
Metastasis status (*N*=7, 12.72%)	
Unilateral lymph node metastasis	3 (5.45%)
Bilateral lymph node metastasis	1 (1.82%)
Distant metastasis	3 (5.45%)

### Pathological results and surgical outcomes

**Fig. 2:**
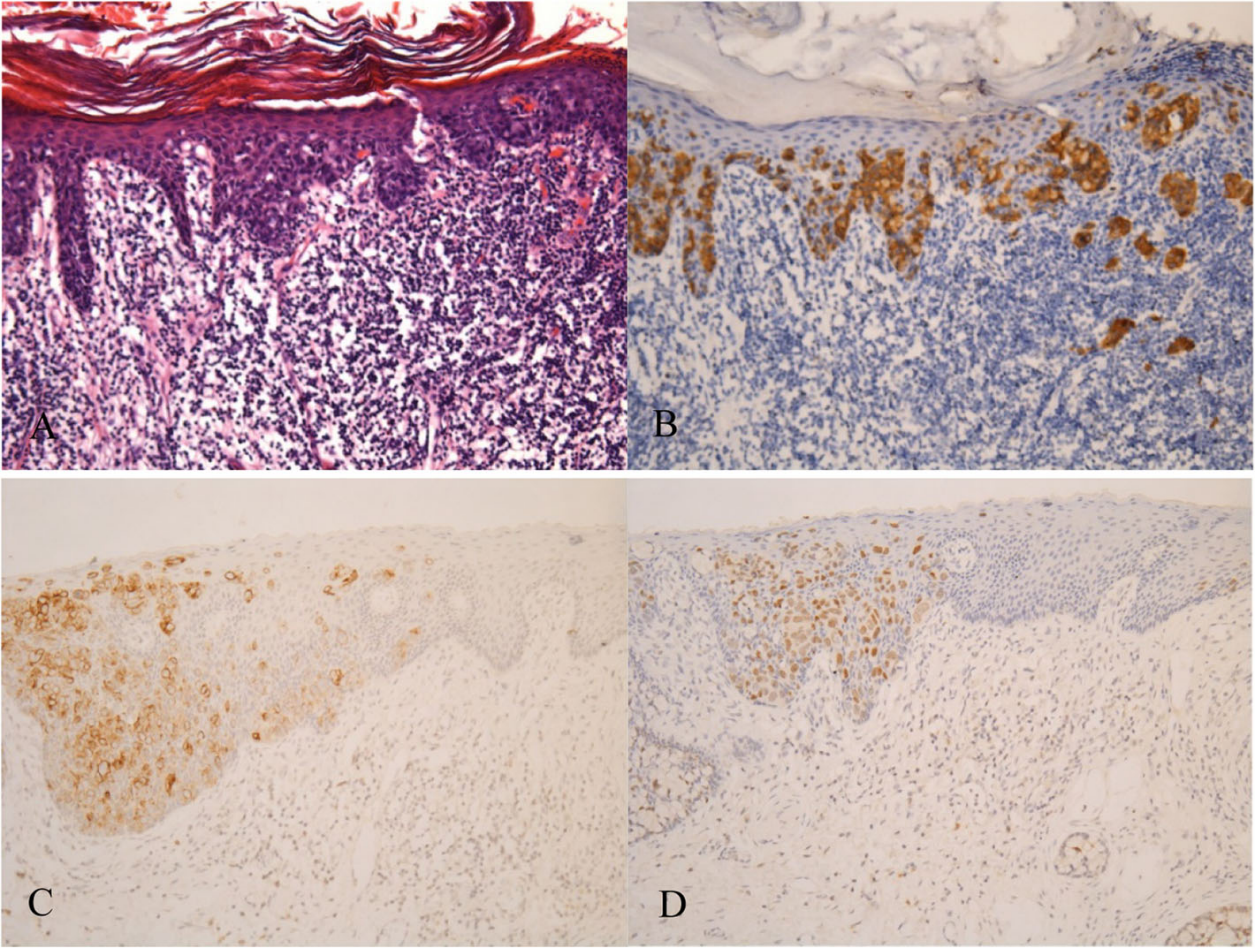
Images of immunohistochemical staining in EMPD of our series. **a** Primary EMPD with micro-invasion ×10. **b** Positive expression of CK7 ×10. **c** Positive expression of CK20 ×10. **d** Positive expression of Cdx2 ×10

Based on the pathological results of preoperative biopsy, among the 55 eligible patients, 17 had invasive lesions (30.91%), including 10 lesions with microinvasion of upper dermis (18.18%) and 7 lesions with deep invasion (12.73%). Due to lack of the detailed staining results in some patient’s electronic record, we analyzed the results of 3 immunohistochemical staining markers among 36 patients, including CK7, CK20, and GCDFP-15 (Fig. 2). Among the 36 immunohistochemically stained EMPD specimen, 33 were positive of CK7 (91.67%), 8 were positive of CK20 (22.22%), 5 were positive of GCDFP-15 (13.89%), and 8 were positive of Cdx2 (22.22%) (Table 1). Seven cases indicated metastasis (12.72%), including three unilateral lymph node metastases (5.45%), one bilateral lymph node metastasis (1.82%), and three distant metastases (5.45%). A total of 47 patients (85.46%) received surgical excision of the lesion, including 8 (14.55%) who received surgical treatment with adjuvant therapy. Four patients (7.27%) received radiotherapy or chemotherapy without surgical excision, whereas another four (7.27%) refused any treatment.

### Overall survival rate and prognostic factors

After diagnosis, the overall survival rate declined over the years (Table 2). The overall survival rate was 80.00% at 36 months and 65.45% at the end of follow-up. Cause-specific hazard model of 5-year all-cause mortality was performed (Table 3) for each variable. Patients with regional metastasis or distant metastasis had poorer prognosis of 5-year survival compared with patients without metastasis (sub-HR 4.513, *p* = 0.0028, CI 1.683–12.103). Furthermore, patients with deep dermal invasion had worse prognosis compared with those without dermal invasion (sub-HR 5.167, *p* = 0.0015, CI 1.876–14.230), whereas no similar harmful effect was noted in the microinvasion of dermis (*p* = 0.6362). No other significant prognostic factor was found among the other variables, including age, anatomic site of lesion, size of lesion, type of treatment, or concurrent malignancy. Proportional hazard assumption was tested, and there was no indication of violating the assumption.

**Table 2:** Five-year overall survival rate and recurrence-free survival rate

Years after diagnosis	Number or survival	Rate
5-year overall survival rate (*N*=55)		
1st year	51	92.73%
2nd year	47	85.45%
3rd year	44	80.00%
4th year	41	74.55%
5th year	36	65.45%
Years after diagnosis	Number of recurrence-free survival	Recurrence-free survival rate
5-year recurrence-free survival rate (*N*=51)		
1st year	46	90.20%
2nd year	38	74.51%
3rd year	36	70.59%
4th year	34	66.67%
5th year	31	60.78%

**Table 3:** Competing risk analysis of 5-year mortality

Variate	Mortality rate	Sub-HR	95% CI	p-value
Gender				
Female	16.67%	**Reference**	**Reference**	**Reference**
Male	39.53%	**2.737**	**0.632–11.859**	**0.1784**
Age				
Age less than 75 years old	24.00%	**Reference**	**Reference**	**Reference**
Age= 75 years old or more	43.33%	**2.127**	**0.808–5.600**	**0.1265**
Lesion site				
Genital region or others	26.32%	**Reference**	**Reference**	**Reference**
Perianal or perineal region	52.94%	**2.338**	**0.948–5.765**	**0.0651**
Size of lesion				
Lesion = 2 cm or less	26.32%	**Reference**	**Reference**	**Reference**
Lesion lager than 2 cm	38.89%	**1.624**	**0.584–4.514**	**0.3527**
Invasion of dermis				
No dermal invasion	28.95%	**Reference**	**Reference**	**Reference**
Micro-invasion	20.00%	**0.695**	**0.154–3.137**	**0.6362**
Deep dermal invasion	85.71%	**5.167**	**1.876–14.230**	**0.0015**
Metastasis status				
Without metastasis	27.08%	**Reference**	**Reference**	**Reference**
Metastatic diseases	85.71%	**4.513**	**1.683–12.103**	**0.0028**
Recurrence (*N*=51)				
No recurrence	27.91%	**Reference**	**Reference**	**Reference**
With recurrence	62.50%	**2.587**	**0.907–7.382**	**0.0756**
Concurrent malignancy				
No concurrent malignancy	29.41%	**Reference**	**Reference**	**Reference**
Adnexal carcinoma	33.33%	**1.180**	**0.151–9.225**	**0.8744**
Internal malignancy	44.44%	**1.595**	**0.629–4.044**	**0.3249**
Types of treatment				
With surgical excision	31.91%	**Reference**	**Reference**	**Reference**
Without surgical excision	50.00%	**1.642**	**0.544–4.950**	**0.3787**
Margin status (*N*=47)				
Margin not free	33.33%	**Reference**	**Reference**	**Reference**
Margin free	31.03%	**0.911**	**0.324–2.561**	**0.8596**
Intraepithelial lesion (N= 31)				
Margin not free	37.50%	**Reference**	**Reference**	**Reference**
Margin free	21.74%	**0.528**	**0.126–2.211**	**0.3822**

*Abbreviatio*n: *CI* confidence interval, *Sub-HR* sub-hazard ratio

### Recurrence rate and 5-year recurrence-free survival

During the 5-year follow-up, among the fifty-one patients who received any type of treatment, eight patients suffered from recurrence (15.69%), with a mean recurrence interval of 15.5 months (range 1.3–29.6 months). The recurrence-free survival rate declined more rapidly over the years than the overall survival rate (Table 2). The recurrence-free survival rate was 70.59% at 36 months and 60.78% at the end of the follow-up. Competing risk analysis with cause-specific hazard model (Table 4) of recurrence in the 5-year follow-up interval showed a similar outcome as the overall survival. Metastatic disease (sub-hazard ratio 9.103, *p* = 0.002, CI 2.249–36.849) and deep dermal invasion (sub-HR 7.836, *p* = 0.0052, CI 1.848–33.449) were significant factors leading to poor outcome of recurrence-free survival. No significant association was observed between the margin status and recurrence (*p* = 0.4338). In the subgroup analysis of those with intraepithelial lesion, free-margin status revealed no significant benefit of recurrence-free survival compared with those without free excision margin (*p* = 0.3998). No other significant risk factor of recurrence was found in other variables. Proportional hazard assumption was tested, and there was no indication of violating the assumption.

**Table 4:** Competing risk analysis of recurrence (*N*=51)

Variate	Recurrence rate	Sub-HR	95% CI	***p***-value
Gender				
Female (*N*=12)	16.67%	**Reference**	**Reference**	**Reference**
Male (*N*=39)	15.38%	**0.884**	**0.178–4.381**	**0.8799**
Age				
Age less than 75 years old (*N*=25)	20.00%	**Reference**	**Reference**	**Reference**
Age= 75 years old or more (*N*=26)	11.54%	**0.673**	**0.161–2.818**	**0.5878**
Lesion site				
Genital region or other (*N*=36)	16.67%	**Reference**	**Reference**	**Reference**
Perianal or perineal (*N*=15)	13.33%	**0.835**	**0.169–4.138**	**0.8253**
Size of lesion				
Lesion = 2 cm or less (*N*=16)	6.25%	**Reference**	**Reference**	**Reference**
Lesion lager than 2 cm (*N*=35)	20.00%	**3.743**	**0.460–30.454**	**0.2171**
Invasion of dermis				
No dermal invasion (*N*=45)	11.11%	**Reference**	**Reference**	**Reference**
Deep dermal invasion (*N*=6)	50.00%	**7.863**	**1.848–33.449**	**0.0052**
Metastasis				
Without metastasis (*N*=44)	9.09%	**Reference**	**Reference**	**Reference**
With any metastasis (*N*=7)	57.14%	**9.103**	**2.249–36.849**	**0.0020**
Concurrent malignancy				
No concurrent malignancy (*N*=32)	12.50%	**Reference**	**Reference**	**Reference**
Adnexal or Internal malignancy (*N*=19)	21.05%	**1.955**	**0.488–7.828**	**0.3436**
Excision margin status (*N*=47)				
Margin not free (*N*=18)	22.22%	**Reference**	**Reference**	**Reference**
Margin free (*N*=29)	13.79%	**0.575**	**0.144–2.300**	**0.4338**
Intraepithelial lesion (*N*= 31)				
Margin not free (*N*=8)	25.00%	**Reference**	**Reference**	**Reference**
Margin free (*N*=23)	13.04%	**0.463**	**0.077–2.779**	**0.3998**

*Abbreviation*: *CI* confidence interval, *Sub-HR* sub-hazard ratio

### Concurrent malignancy

Concurrent or subsequent malignancy was noted in 21 patients (38.18%), including 3 patients (5.45%) with adnexal carcinoma and 18 patients (32.73%) with internal malignancy (Table 1). Among the 18 patients with internal malignancy, 10 were diagnosed with gastrointestinal tract malignancy, 4 with genitourinary tract malignancy, 2 with adenocarcinoma with unknown origin, and 2 with parotid cancer. When analyzed with anatomic site of lesion, among 17 patients with perianal EMPD, 8 patients were diagnosed with gastrointestinal tract malignancy (47.06%) compared with 2 gastrointestinal tract malignancy in 38 patients with EMPD (5.26%) in other sites. In the 29 patients with genitourinary EMPD, 3 patients with genitourinary tract malignancy was observed (10.34%), whereas a genitourinary tract malignancy was detected in the other 26 EMPD patients (3.85%). Logistic regression of EMPD anatomic site and internal malignancy revealed the strong association between gastrointestinal malignancy and perianal region EMPD (odds ratio = 16.00, *p* = 0.0015, CI 2.885–88.730), whereas no similar association was noted in genital region EMPD and genitourinary malignancy (*p* = 0.3726) (Table 5).

**Table 5:** Logistic regression analysis of EMPD and internal malignancy

Variate	Odds ratio	95% confidence interval	*p*-value
Perianal EMPD and gastrointestinal malignancy	16.000	2.885–88.730	0.0015
Genital region EMPD and genitourinary malignancy	2.884	0.281–29.609	0.3726

## Discussion

In the present study, the characteristics of EMPD patients in one single institution were analyzed. As revealed in other Asian population-based studies [7, 12], the predominance of male gender in the distribution of EMPD patients was also noted in our cases. The most common affected site was the penoscrotal region (40%), similar to the findings of other studies [13–15]. The average size of lesion, the mean age of diagnosis, and rate of metastasis (12.72%) were also in compatible range with previous literature [4, 5, 7, 12]

The overall survival rates in our study were 80.00% (36-month follow-up) and 65.45% (60-month follow-up) (Table 2), which were compatible with those of previous male-predominant or Asian-predominant study [12, 13]. Previous studies had identified several potential risk factors of poor prognosis of EMPD, including the level of tumor invasion, lymph node metastases, elevated CEA, perianal lesion, old age, and male gender [5, 12, 13, 16]. In our study, based on the results of cause-specific hazard model (Table 3), metastatic diseases and deep dermal invasion were identified as significant harmful factors of the overall 5-year survival, showing similar outcomes with two population-based studies and previous reviews [6, 12, 17]. The relationship between survival and microinvasive disease remains controversial, whereas deeply invasive EMPD was linked to poorer prognosis than the non-invasive counterpart [12, 17]. The association between prognosis and site of lesion had been reported, suggesting that anorectal EMPD has a statistically significantly decreased mean disease-specific survival compared with those without anorectal involvement [4]. However, no significant difference in overall survival was observed between the different groups of lesion site in our study (Table 3).

The 5-year recurrence rate (15.69%) and the mean recurrence interval (15.5 months after diagnosed) in our study were similar to those of other EMPD studies that treated patients with wide local excision [14, 15, 18]. The recurrence-free survival rate was 70.59% at 36-month follow-up and 60.78% at 60-month follow up (Table 2), consistent with those of other wide local excision studies [15, 18]. Based on the results of competing risk analysis (Table 4), metastatic diseases and deep dermal invasion were identified as potential risk factors of recurrence. The results of our study coincided with that of a previous study [10], whereas another population-based study reported no relationship between dermal invasion and local recurrence [7]. Previous literature observed a strong association between margin status and recurrence risk [15], whereas in our study (Table 4) no similar significant association was found. In the subgroup analysis of those with intraepithelial lesion, free-margin status revealed no improvement in recurrence-free survival compared with those without free excision margin (*p* =0.3998), which was in conflict with previous literature [19].

The rates of concurrent malignancy (38.18%), adnexal carcinoma (5.45%), and internal malignancy (32.73%) in our study were in compatible range with previous reviews [2, 5, 15, 20]. Several Asian population-based studies revealed a low concurrent internal malignancy rate in Asian EMPD patients [7, 12, 14] which is in contrast with the result of our study. The potential relationship between the anatomic site of EMPD lesion and internal malignancy was proposed in another study [20]. We determined the perianal EMPD as a significant risk factor of gastrointestinal malignancy (odds ratio = 16.00, *p* = 0.0015, CI 2.885–88.730), whereas no similar association was observed between the genital region EMPD and genitourinary malignancy (*p* = 0.3726) (Table 5).

Our study had several limitations. First, all the data were retrospectively extracted from the electronic patient record system, which may lead to potential bias in data extraction or misinterpretation. Inadequate description of pathology reports and outpatient department follow-up may also lead to underestimation of the actual rate of dermis invasion and recurrence. In addition, given the long follow-up period of up to 5 years, phone interview was performed as an alternative way of evaluation, in which only limited information can be accessed. Finally, with the rarity of EMPD in Asian population, the present single-center study included 55 illegible patients. A multicenter, larger sample size study in Taiwanese population is still needed for further evaluation.

To the best of our knowledge, this research is the first study in the English language literature about the comprehensive survival analysis of EMPD in Taiwan population. Our report also identified similar disease characteristics and prognostic factors in Taiwan population, similar to other Asian population-based studies, and their differences.

## Conclusion

EMPD is commonly observed among aged people. The presence of metastatic EMPD and deep dermal invasion are significant harmful factors of the overall 5-year survival and 5-year recurrence-free survival. In most cases, EMPD is not associated with cancer, whereas perianal EMPD is accompanied with a high risk of gastrointestinal malignancy. Regardless of treatment method, long-term follow-up is recommended.

## Data Availability

The datasets used and/or analyzed during the current study are available from the corresponding author on reasonable request.

## Abbreviations

EMPD: Extramammary Paget’s disease
sub-HR: Sub-hazard ratio
